# Glances at the Hospitals

**Published:** 1901-05-04

**Authors:** 


### GLANCES AT THE HOSPITALS.

### THE MIDDLE-WARD HOSPITAL FOR LANARK.

This hospital for fever cases was opened in October 1897, and it has since admitted a total of 1,594 cases. In the year 1899 there were at the beginning of the year 114 patients in residence, and during the year 849 cases were admitted. Two hundred and thirty-five of these were cases of enteric fever, 571 of scarlet fever, 1 of measles, 21 of diphtheria, 5 of erysipelas, 4 of other diseases, and 12 were kept in quarantine. Since the opening of the hospital enteric fever has shown a mortality of 11·6 per cent., scarlet fever of 3·7 per cent., measles of 18·5 per cent., diphtheria of 12·9 per cent., and erysipelas of 28·5 per cent. In 1899 there were 21 fatal cases of enteric fever, and these presented the following complications:—Perforation 1, intestinal hæmorrhage 10, general hæmorrhage 1, bronchitis 5, pneumonia 3, nephritis 1, ostitis 2, hemiplegia 1. Dr. Buchan points out once more the extraordinary variable nature of enteric fever, and it is not surprising to hear that 36 were notified to him as enteric fever, and turned out not to be that disease at all. On the other hand, three patients were found to be enteric, although not so certified on admission. The average duration of the disease in the recovered cases was 45 days; in the fatal cases it was 16 days. Taking the 201 recovered cases we learn that diarrhœa was present in 78 cases and constipation in 63; the rose-coloured spots were seen in 145 cases, but in this connection it has to be remembered that 80 of the cases did not come under observation until the third week of the disease; other eruptions were present in seven cases, of which three were scarlatiniform, and in the rash was so like that of scarlatina that the case was notified as that disease. Distinct splenic enlargement was present in 121 cases. In 14 cases relapses took place. In these the mean duration of the primary attack was 25 days, the apyrexial period ten days, and secondary attack 14 days. All these cases recovered. Bronchitis was noted in 33 instances, pneumonia in 7, pleurisy in 1, nephritis in 9, otitis media in 10, insanity in 2, venous thrombosis in 3, hemiplegia in 1, and ulceration of the larynx in 1. We have entered thus fully into the statistics of enteric fever from the extreme importance of the disease, but it should be stated that particulars concerning the other diseases are stated with equal clearness in the report. The average daily number of patients resident was 115, and for these the following staff is provided: One physician, one matron, two ward sisters, seven trained nurses, eleven probationers, one sewing-maid, one cook, one kitchenmaid, three laundry-maids, two housemaids, and four wardmaids. We trust we shall not be considered captious if we suggest that next year's report should contain a table showing the average cost per patient and the average cost per week, such sums to include everything excepting what may be spent on the building.

### THE LIVERPOOL STANLEY HOSPITAL.

The expenditure for 1899 was £4,344, and the income, exclusive of legacies, was £3,816; but this sum includes over £1,000 from donations and entertainments. At the beginning of the year there was an adverse balance of £1,865, and this has only been reduced by about £30. The general statistics are not very well arranged. We learn that the in-patients numbered 1,220, and the out-patients 17,148; but so far as the in-patients are concerned we failed to notice any statement of the cost per bed occupied, or the cost per head per week, or the average residence in the hospital. There is a table showing that 406 major surgical operations were performed, and the diseases which necessitated the operations and the results are given. So far so good. But when we turn to the other medical and surgical tables we can find only the names of the diseases and the number of patients suffering from each, but no results; and there is no mention of the kind of anæsthetics used in the operations.

### THE EVERSFIELD HOSPITAL FOR DISEASES OF THE CHEST, ST. LEONARDS-ON-SEA.

The total income for the year 1899 was £1,852, and the expenditure was within a few shillings of that sum. The in-patients' payments amounted to £1,384; out-patients' payments to £43; subscriptions to £117; donations, £269. The hospital can accommodate 55 patients, including provision for 8 private cases. Patients are received from all parts of the country, irrespective of creed or nationality. During 1899, 242 patients were admitted and 30 remained at the end of the year. The report states that there is a steadily increasing number of patients attending the out-patients' department, and it is added that "it is free to the necessitous poor, and many take advantage of it; but in order to avoid the pauperising effect of gratuitous advice it is expected that everyone who is able should give something, however small, to help meet the expenses." The report is presented to us innocent of all medical details. We think this is an unfortunate omission.

THE BURY DISPENSARY HOSPITAL.

The total income amounted to £3,250, and the expenditure exceeded this by £330. At the beginning of 1899 the hospital contained 27 patients, and 376 were admitted during the year. Of the total of 403, the recoveries were 304; the discharged relieved, 39; the deaths, 27; and 33 remained under treatment. It should be noted that 10 of the deaths took place within 12 hours of admission, and 6 within 48 hours. Deducing these 16 cases, the rate of mortality was 2·75 per cent. The vast majority of the patients were surgical cases; and anæsthetics were administered 158 times. The average stay of the patients in the hospital was 28 days. There were 1,020 out-patients. The medical and surgical statistics are imperfect, as no results of treatment are shown. It is, for instance, useless to be told that there were 16 cases of hernia and 6 cases of trephining the skull if we hear nothing of termination of the treatment on operations.

